# Comparison of Stimulus Types for Retinotopic Cortical Mapping of Macular Disease

**DOI:** 10.1167/tvst.12.3.6

**Published:** 2023-03-13

**Authors:** Maximilian Pawloff, David Linhardt, Michael Woletz, Allan Hummer, Stefan Sacu, Maria Vasileiadi, Lerma Usabiaga Garikoitz, Graham Holder, Ursula M. Schmidt-Erfurth, Christian Windischberger, Markus Ritter

**Affiliations:** 1 Department of Ophthalmology, Medical University of Vienna, Vienna, Austria; 2 MR Center of Excellence, Center for Medical Physics and Biomedical Engineering, Medical University of Vienna, Vienna, Austria; 3 BCBL Basque Center on Cognition, Brain and Language Donostia, San Sebastian, Gipuzkoa, Spain; 4 IKERBASQUE Basque Foundation for Science, Bilbao, Spain; 5 Department of Ophthalmology, Yong Loo Lin School of Medicine, National University of Singapore, Singapore; 6 UCL Institute of Ophthalmology, London, UK

**Keywords:** functional magnetic resonance imaging (fMRI), population receptive field (pRF), geographic atrophy (GA)

## Abstract

**Purpose:**

Retinotopic maps acquired using functional magnetic resonance imaging (fMRI) provide a valuable adjunct in the assessment of macular function at the level of the visual cortex. The present study quantitatively assessed the performance of different visual stimulation approaches for mapping visual field coverage.

**Methods:**

Twelve patients with geographic atrophy (GA) secondary to age-related macular degeneration (AMD) were examined using high-resolution ultra-high field fMRI (Siemens Magnetom 7T) and microperimetry (MP; Nidek MP-3). The population receptive field (pRF)-based coverage maps obtained with two different stimulus techniques (moving bars, and rotating wedges and expanding rings) were compared with the results of MP. Correspondence between MP and pRF mapping was quantified by calculating the simple matching coefficient (SMC).

**Results:**

Stimulus choice is shown to bias the spatial distribution of pRF centers and eccentricity values with pRF sizes obtained from wedge/ring or bar stimulation showing systematic differences. Wedge/ring stimulation results show a higher number of pRF centers in foveal areas and strongly reduced pRF sizes compared to bar stimulation runs. A statistical comparison shows significantly higher pRF center numbers in the foveal 2.5 degrees region of the visual field for wedge/ring compared to bar stimuli. However, these differences do not significantly influence SMC values when compared to MP (bar <2.5 degrees: 0.88 ± 0.13; bar >2.5 degrees: 0.88 ± 0.11; wedge/ring <2.5 degrees: 0.89 ± 0.12 wedge/ring; >2.5 degrees: 0.86 ± 0.10) for the peripheral visual field.

**Conclusions:**

Both visual stimulation designs examined can be applied successfully in patients with GA. Although the two designs show systematic differences in the distribution of pRF center locations, this variability has minimal impact on the SMC when compared to the MP outcome.

## Introduction

Retinotopic mapping of the visual cortex based on functional magnetic resonance imaging (fMRI) acquired with blood oxygenation level-dependent (BOLD) contrast reveals the systematic representation of visual space in the visual cortex.^1^ Retinotopic organization describes a feature of the visual system whereby every point on the retina corresponds to a specific point inside the visual cortex. The single-neuron response on the cortex to a visual stimulus is called a receptive field. It is not possible to measure activation at a single-neuron level due to limitations in fMRI resolution. The clustered activation within a three-dimensional measurement region corresponds to a population of neurons, referred to as a population-receptive field (pRF). Visual stimuli moving through the subject's field of view in a known manner excite specific patterns in the visual cortex and allow for the reconstruction of the retinotopic organization.^2^^,^^3^ It is possible to determine these pRFs in vivo using the advanced computational neuroimaging approach of pRF mapping.

Previous studies have shown that pRF mapping provides objective functional data with high concordance to conventional testing, including microperimetry and optical imaging in both patients with central or peripheral retinal scotomata and healthy controls with artificial scotomata.^4^^,^^5^ Other studies investigating retinal diseases^6^^–^^9^ or retrochiasmal visual pathway lesions^10^ have shown similar results. A combination of pRF mapping with conventional testing in patients with retinal dysfunction allows for a more objective assessment of visual function as bias from attention level changes is minimized, of particular importance for longitudinal studies aimed at assessing treatment effects in novel therapeutic interventions. This was recently shown in patients with RPE65-associated retinal dystrophy who, following gene replacement therapy, demonstrated widespread cortical activation in areas with an undetectable cortical response prior to treatment.^11^

The standard setup for pRF mapping is based on visual stimuli resembling either rotating wedges and expanding/contracting rings as stimulus patterns^12^^,^^13^ or bar apertures moving through the visual field in different directions, both revealing an isoluminant reversing checkerboard.^3^ Although different variants of similar stimuli have been used, for example, landscapes, textures, animals, or faces,^14^ either bar or wedge/ring stimuli remain the prime pRF mapping approaches.^15^^–^^17^ Fundamental to any future clinical utility of pRF mapping is the assessment of whether the choice of visual stimulation patterns might bias pRF mapping results in patients suffering from retinal disease.

Geographic atrophy (GA), a common feature of macular disease, is characterized by sharply demarcated central macular atrophy and central visual field loss. Macular lesions may initially appear perifoveally and expand over a number of years to involve the fovea.^18^ This distinct lesion pattern makes patients with GA prime subjects for exploring the cortical representation of central retinal scotomata. The method of pRF mapping is perfectly suited for the measurement of scotomata yielding very high reproducibility values,^19^ even though biases in the modeling results are introduced.^20^

The present study utilizes a crossover design to compare pRF maps obtained from wedge/ring stimulation to those from bar stimulation in patients with GA secondary to age-related macular degeneration (AMD).

## Patients and Methods

Twelve patients with GA (8 men and 4 women; age = 72.6 ± 5.1 years) were recruited and underwent fMRI measurements. Written informed consent was obtained from all subjects before their participation. All patients had a secure clinical diagnosis supported by optical imaging studies. Inclusion criteria were a central, well-demarcated atrophic macular lesion; a central scotoma not exceeding 15 degrees visual angle diameter and fixation stability classified as stable or relatively unstable as measured by microperimetry. No patients with preferred retinal locus (PRL) were included. The study was approved by the local ethics committee.

### Clinical Examination

Patients underwent a full ophthalmic examination, including slit-lamp examination and dilated fundus examination, fundus autofluorescence imaging, optical coherence tomography, and microperimetry. Best corrected visual acuity (BCVA) was measured using Early Treatment Diabetic Retinopathy Study (ETDRS) charts.

### Retinal Imaging

Spectral-domain optical coherence tomography (SD-OCT) and blue-light fundus autofluorescence (FAF) images were recorded using a Spectralis HRA and OCT system (Heidelberg Engineering, Heidelberg, Germany) to evaluate retinal structure.

### Microperimetry

Macular function was assessed by an MP-3 microperimeter (MP; Nidek, Padova, Italy). Stimulus intensity ranged from 0 dB to 32 dB in 1 dB steps, with the initial intensity at 17 dB. The stimulus pattern consisted of a foveal 3 × 3 grid surrounded by 3 rings at a radius of 3 degrees (8 points), 5.1 degrees (12 points), and 7 degrees (12 points) eccentricity. MP was measured at the anatomic fovea. Fixation stability was assessed as part of the microperimetric examination. Fixation was classified as stable when 90% of fixations were located within a 2 degrees circle, as relatively unstable when ≥80% of fixations were located within a 2 degrees circle and as unstable when less than 80% of fixations were located within a 2 degrees circle.

### Functional MRI Measurements

Functional MRI measurements were performed using a 32-channel head coil in an ultra-high field Siemens MAGNETOM 7T scanner (Siemens Healthineers, Erlangen, Germany). Subjects participated in one scanning session including four functional runs, acquired using the CMRR EPI sequence^21^ at an isotropic spatial resolution of 1 mm, matrix size = 128 × 128; TR/TE = 2000/25.2 ms; GRAPPA = 2; slice spacing = 10%. Every run included 32 slices covering the subject's visual cortex, placed perpendicular to the calcarine sulcus. Further, a structural full-brain image was obtained using a magnetization-prepared rapid gradient-echo (MPRAGE) sequence with 0.7 mm isotropic resolution. Each scanning session lasted approximately 1 hour.

Stimuli were presented via a back-projection screen mounted at the end of the patient’s bed. Subjects viewed the screen through a mirror attached to the head coil with a mean distance between the eyes and the screen of 62 cm. If both eyes were affected by GA, the study eye was chosen randomly. One eye was patched to focus the measurement on eye-specific pathology. Subject motion was restricted by extensive padding.

Preprocessing of the functional data was performed in a custom-built pipeline (Matlab, SPM, and FSL) including slice-timing correction, re-alignment, distortion correction, and spatial smoothing using a Gaussian kernel with FWHM of 2 mm. The anatomic image was segmented using Freesurfer (https://surfer.nmr.mgh.harvard.edu) to obtain white and grey matter masks and manually corrected for segmentation and topological errors.

The two measured runs per session were averaged before analysis to improve the signal-to-noise ratio. The pRF analysis was performed using the Matlab-based toolbox mrVista (https://github.com/vistalab/vistasoft). Within this analysis, time-course models are created based on different positions on the visual field (x and y) and receptive field sizes. For every voxel inside the participant's visual cortex, the best-fitting model is determined and a discrete mapping between the position on the visual field and the cortex is established. All pRF analyses were performed without any information on the scotoma status of the subjects studied. All reported data are thresholded at 20% variance explained.

### Stimuli

**Figure 1.**
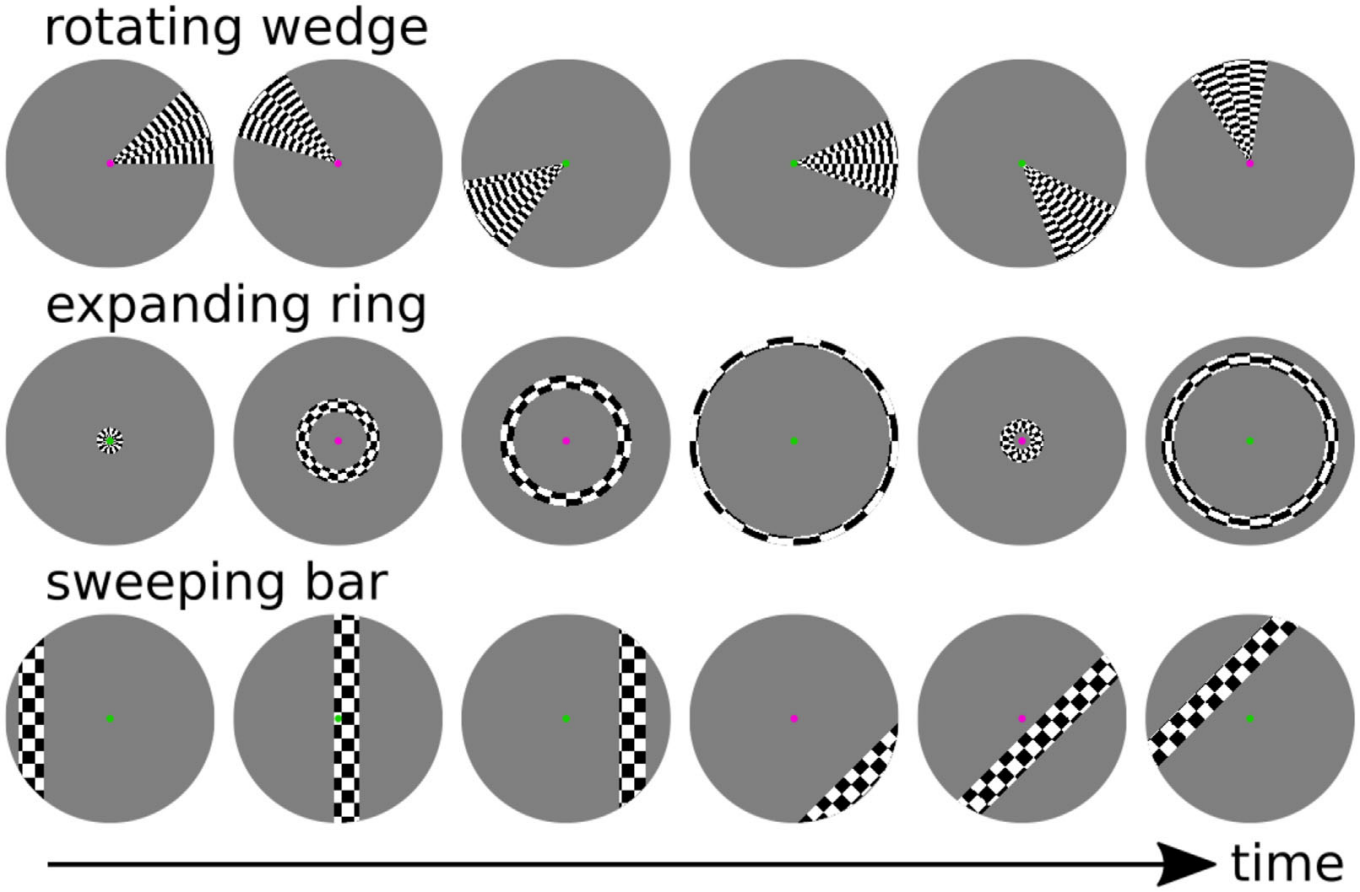
Representative images from bar and wedge/ring stimuli.

Stimulation patterns during the fMRI examination covered the central 14 degrees visual angle and were presented using mrVista (Vista Lab, Stanford University, Stanford, CA) within the Matlab programming environment (The MathWorks, Inc., Natick, MA). Two stimulus designs, “bar” and “wedge/ring,” were examined (see Fig. 1).

The first stimulus consisted of a bar moving across an isoluminant screen while exposing a checkerboard pattern reversing with a frequency of 8 Hz. Bar width was 1.75 degrees, corresponding to 12.5% of the total stimulus coverage, and crossed the screen in 18 discrete steps, each separated by 0.8 degrees visual angle in space and TR = 2 seconds in time. The bar moved across the screen in eight different directions for each run. After each crossing, the bar was rotated by 45 degrees. With pauses of 12 seconds duration after each diagonal pass, during which the subject was presented a blank grey screen of similar mean luminance, a single-run length was 5 minutes 36 seconds or 168 volumes. Run length was identical for both stimulus variants.

The second stimulus consisted of a counter-clockwise rotating wedge, with a width of 45 degrees and a step size of 20 degrees, performing two full rotations in 36 steps and a ring with a thickness of 0.875 degrees visual angle, expanding from the center twice in 36 steps (step size 0.43 degrees). This whole sequence was repeated in opposite directions (i.e. clockwise rotating wedge and contracting ring). Between each wedge or ring period, the grey background image was shown for 12 seconds as a baseline. The exposed checkerboard is radially symmetrical.

Patients were instructed to fixate on a small dot (12 pixels or 0.22 degrees visual angle diameter) in the center of the screen. As good fixation is essential, thin diagonal lines (5 pixels or 0.09 degrees visual angle diameter) crossing at the center dot were also displayed to assist patients to maintain stable fixation. The color of the fixation dot and cross was changed pseudo-randomly, and subjects were asked to report color changes via a button press. This measure was used to assess subject compliance.

### Correlation of Visual Field Coverage and Microperimetry

Coverage maps were created by plotting the maximum surface of all above-threshold pRFs resulting in a map of the visual field ranging between 0 and 1. Following the study by Ritter et al.,^4^ pRF coverage values above a threshold of 0.7 were classified as areas with good vision and everything below as scotomata.

For comparison, MP results were binarized such that all measurement points with values higher than 0 were classified as areas with vision, whereas 0 was classified as scotomatous. The most peripheral ring of the MP measurement points was not taken into consideration as it fell outside of the area stimulated during the fMRI measurements.

MP and pRF maps were compared on a subject-by-subject basis based on the binarized coverages. The simple matching coefficient (SMC) was used to compare both maps quantitatively within the range between 0 (all point values differ) and 1 (all point values are identical).

$$\begin{array}{c} SMC=\;\frac{\#\;matching\;points}{\#\;points} \end{array}$$

**Figure 2.**
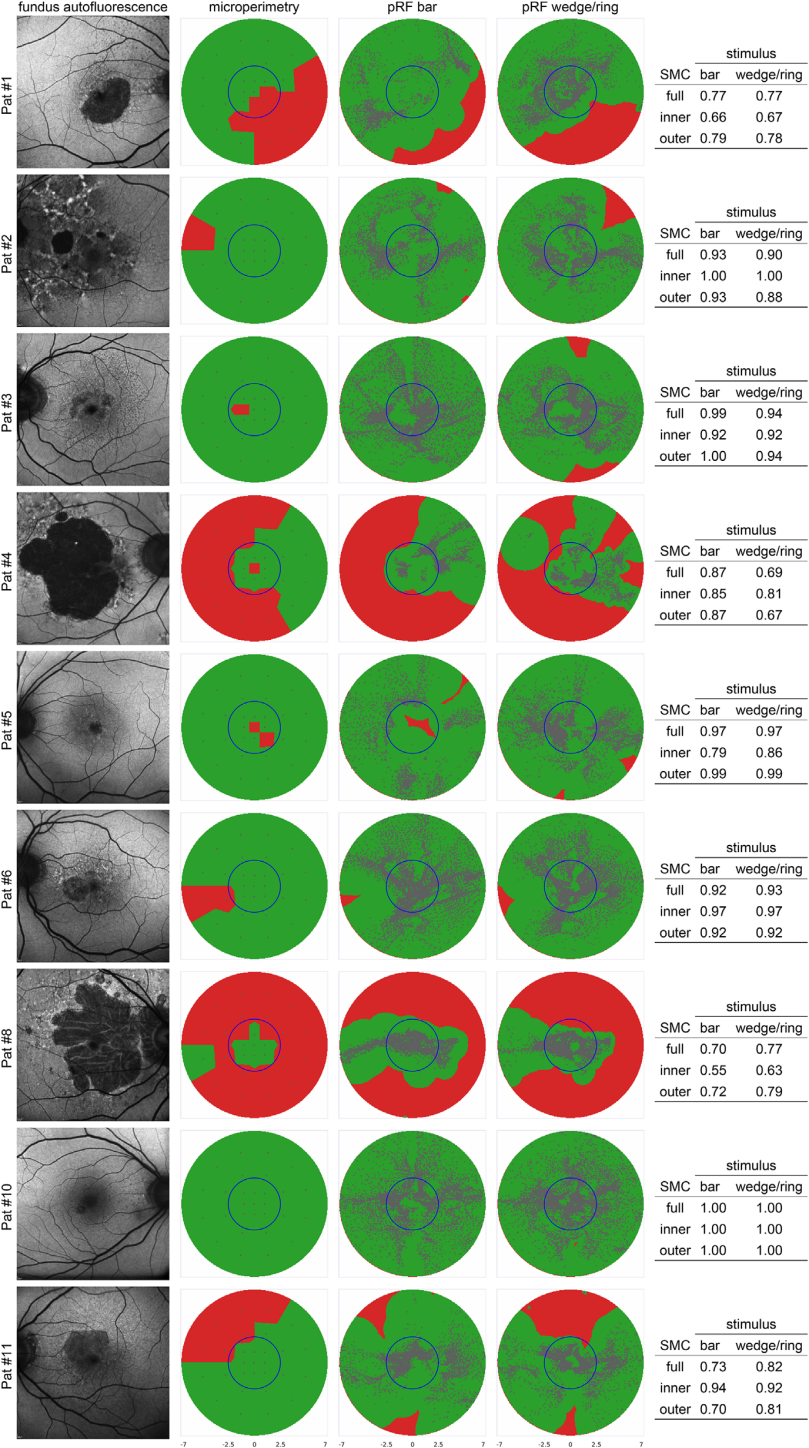
Comparison of FAF, MP, and pRF mapping results for all patients. FAF results (*first column*) show areas of hypoautofluorescence consistent with outer retinal loss. Note that FAF images cover a wider area and are included for orientation only. *Columns 2*, *3*, and *4* show the binarized MP results (*green*: dB >0, and *red*: dB = 0), and the binarized pRF coverage maps (*green*: >0.7; and red: ≤0.7) with the pRF centers overlaid as grey dots (*third column*: bar stimulus; *fourth column*: wedge/ring stimulus). Each dot represents the center of a receptive field of a single voxel obtained from pRF mapping using either bar or wedge/ring stimuli. The tables on the right show SMC values for full, inner, and outer visual field of view regions.

In addition to the full field of view, SMC was calculated also for areas with low eccentricity (<2.5 degrees) and high eccentricity (>2.5 degrees).^22^ Both maps and the SMC appear in Figure 2.

### Statistical Comparison

To investigate possible systematic biases in pRF parameters between the two stimulus variants, we calculated differences in eccentricity, polar angle, pRF size, and variance explained per subject. These differences were averaged per subject and submitted to one-sample *t*-tests to test for stimulus-specific differences across subjects.

## Results

The clinical characteristics of the patient cohort are summarized in Tables 1 and 2. Three of the 12 patients with GA failed to meet data quality criteria due to poor performance during the fMRI runs (reporting changes in fixation dot color or excessive movement) or MP fixation stability. For all patients, pRF analyses yielded the expected patterns of eccentricity, polar angle, and pRF sizes.

**Table 1.** Patient Data

Patient Number	Sex	Age	Measured Eye	VA logMAR	Eccentric Fixation
***GA01***	f	73	OD	0.204	No
***GA02***	f	66	OS	0.097	No
***GA03***	m	71	OS	0.398	No
***GA04***	m	74	OD	0.204	No
***GA05***	m	68	OS	0.498	No
***GA06***	m	77	OS	0.204	No
***GA07***	f	73	OD	0.301	No
***GA08***	m	77	OD	0.301	No
***GA09***	f	64	OS	0.204	No
***GA10***	m	79	OD	0	No
***GA11***	m	69	OS	0.097	No
***GA12***	m	81	OS	0.097	No

**Table 2.** Microperimetry Stability Data

	% MP1 Fixation 2 Degrees	% MP1 Fixation 4 Degrees	MP Fixation Stability	False Positives	False Negatives
***GA01***	81.2	90.8	Relatively unstable	0/2	1/10
***GA02***	94.8	97.5	Stable	0/4	0/10
***GA03***	88.8	98.3	Relatively unstable	0/4	1/5
***GA04***	93.4	99.3	Stable	0/3	2/9
***GA05***	100	100	Stable	0/7	1/6
***GA06***	87.9	97.9	Relatively unstable	0/8	0/4
***GA07***	85.9	95.7	Relatively unstable	0/4	1/9
***GA08***	88.6	98.1	Relatively unstable	0/1	3/9
***GA09***	93.7	97.1	Stable	0/1	2/2
***GA10***	97.4	99.6	Stable	0/7	0/5
***GA11***	82.7	95	Relatively unstable	0/1	0/7
***GA12***	77.9	97.6	Unstable	0/7	0/2

Figure 2 shows a comparison of the data derived from FAF, MP, and pRF mapping for all patients.

Close inspection of the data show differences between pRF maps created from wedge/ring and bar stimulus fMRI runs. Above-threshold pRF centers are generally located more toward the center of the visual field with wedge/ring stimuli than with bar stimuli.

**Figure 3.**
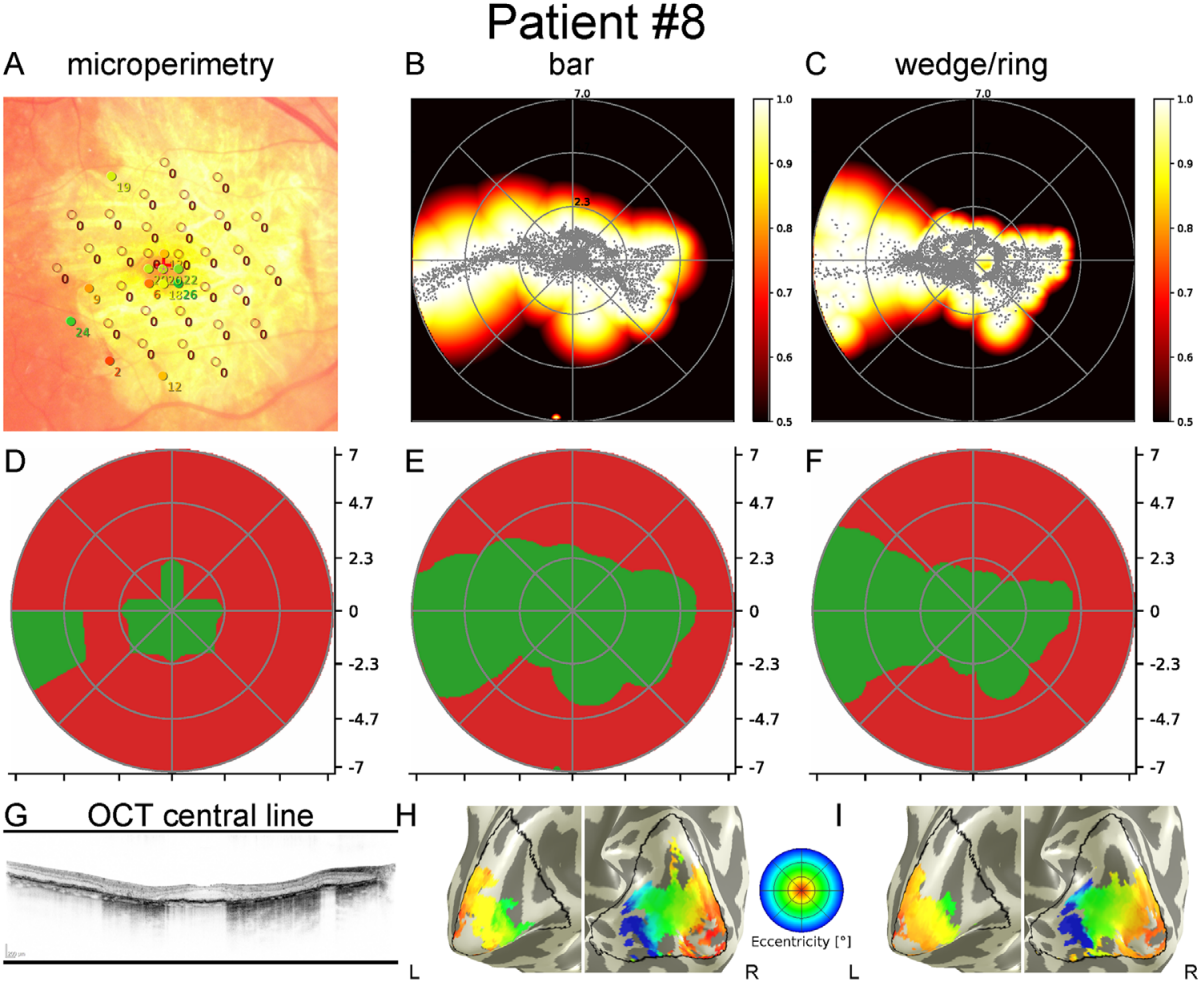
Results of the right eye of patient 8 with fovea-sparing geographic atrophy. *Top row*: MP overlaid on fundus image (**A**) (point colors *red* to *green* indicate 0 dB to 34 dB MP results); pRF coverage maps from bar (**B**) and wedge/ring stimuli (**C**). *Middle row*: Binarized MP (**D**); binarized pRF coverage maps from bar (**E**) and wedge/ring stimuli (**F**). *Bottom row*: Macular OCT (**G**); eccentricity maps from bar (**H**), and wedge/ring stimuli (**I**) overlaid on white matter surface mesh. Preserved central visual function, spanning about 3 degrees visual angle, is clearly visible. Analysis shows no above-threshold voxels representing peripheral areas of the visual field, corresponding to the atrophic areas of the retina while emphasizing the small bridge of preserved outer retina remaining temporally which is not sampled due to MP's discrete grid. A comparison of visual field maps of bar versus wedge/ring stimuli shows differences in pRF center distribution (bar: <2.5 degrees: 3250 [73%], >2.5 degrees: 1178 [27%]; wedge/ring: <2.5 degrees 4011 [85%], and >2.5 degrees: 726 [15%]).

**Figure 4.**
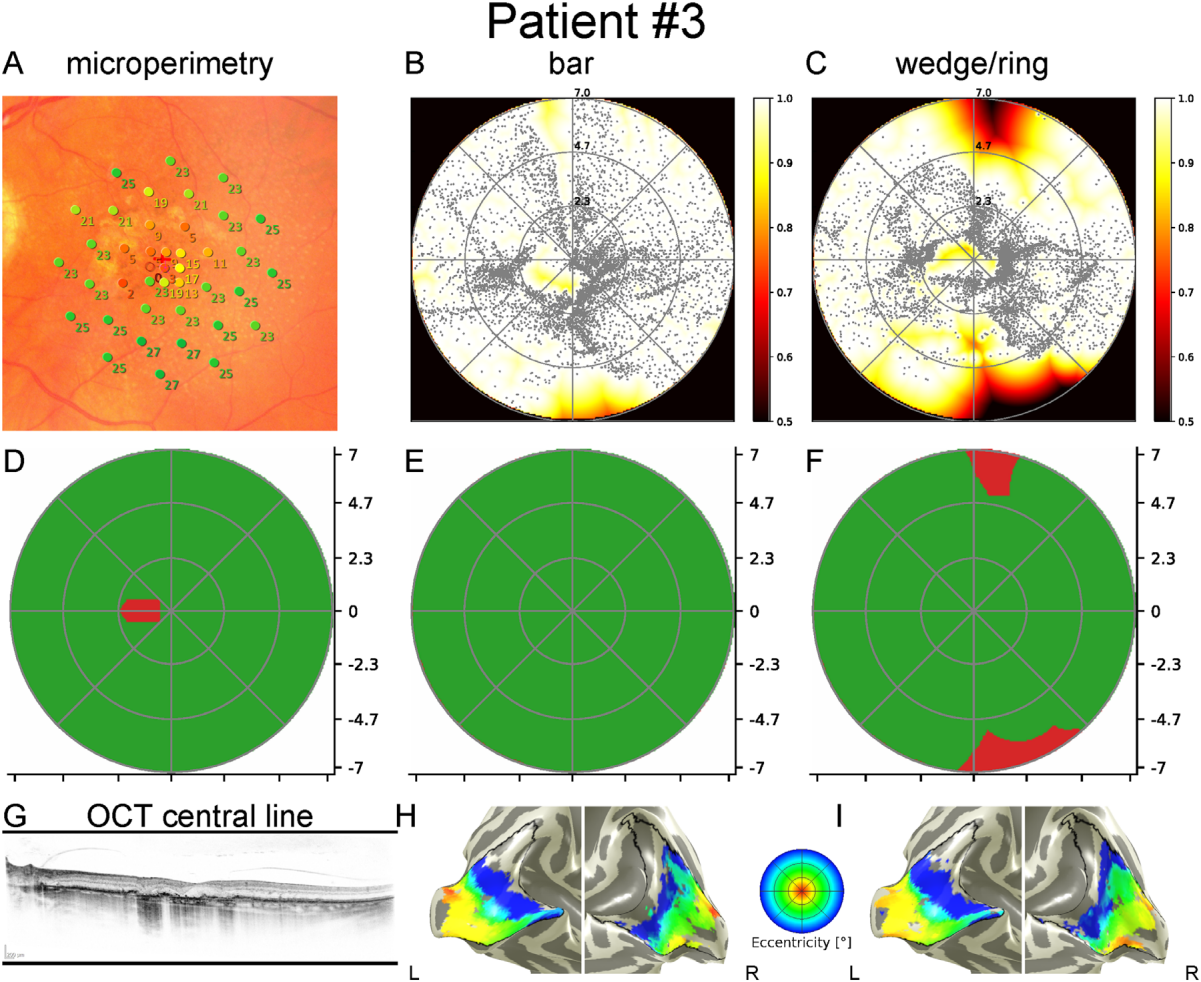
Results of the left eye of patient 3 with geographic atrophy in the superonasal macula. *Top row*: MP overlaid on fundus image (**A**) (point colors *red* to *green* indicate 0 dB to 34 dB MP results); pRF coverage maps calculated from the functional MRI data with bar (**B**) and wedge/ring stimuli (**C**); *Middle row*: Binarized MP (**D**); binarized pRF coverage maps from bar (**E**) and wedge/ring stimuli (**F**); *Bottom row*: Central OCT (**G**); eccentricity maps from bar (**H**) and wedge/ring stimuli (**I**) overlaid on the white matter/grey matter surface mesh. Although the paracentral scotoma is clearly delineated in the distribution of pRF centers on visual field maps, it is not visible in the binarized pRF maps. In the pRF map based on wedge/ring stimuli, the central clustering of pRF centers introduces supposed peripheral visual field defects. There is a clear difference in the distribution of pRF centers between stimuli; pRF centers are distributed homogenously throughout the visual field in visual field maps derived from bar stimuli, but in visual field maps derived from wedge/ring stimuli, they are clustered toward the center (bar: <2.5 degrees: 2640 [40%], >2.5 degrees: 3910 [60%]; wedge/ring: <2.5 degrees 2989 [50%], and >2.5 degrees: 2943 [50%]).

**Figure 5.**
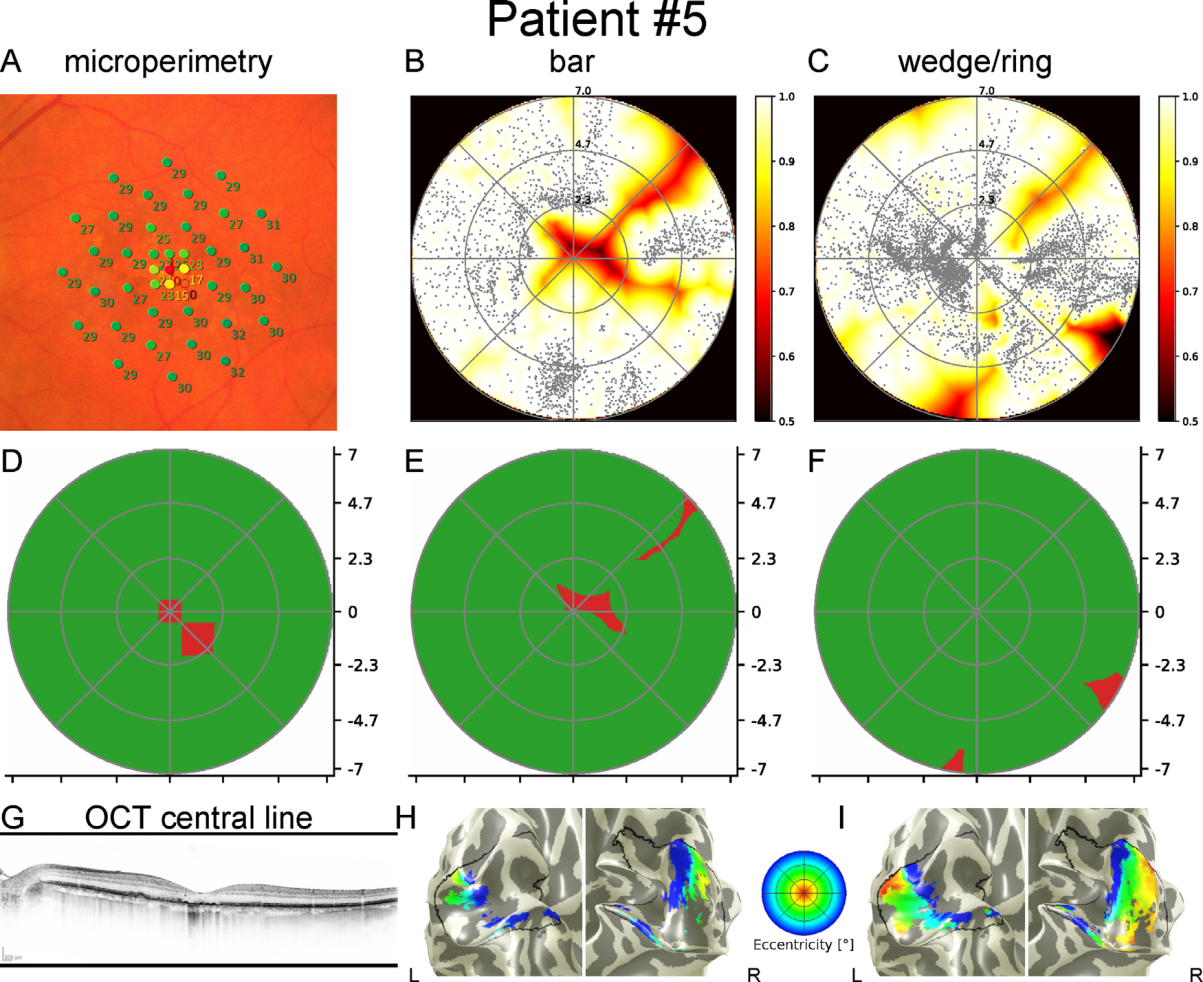
Results of the left eye of patient 05 with a small parafoveal scotoma secondary to geographic atrophy. *Top row*: Superposition of MP on the fundus image where macular sensitivity loss is visible (**A**) (point colors *red* to *green* indicate 0 dB to 34 dB MP results); pRF coverage maps calculated from the functional MRI data with bar (**B**) and wedge/ring stimuli (**C**); *Middle row*: binarized MP (**D**); binarized pRF coverage maps from bar (**E**) and wedge/ring stimuli (**F**). *Bottom row*: Central OCT (**G**); eccentricity maps from bar (**H**) and wedge/ring stimuli (**I**) overlaid on the white matter/grey matter surface mesh. There is a strong agreement between the pRF map based on bar stimuli and MP. However, in the maps based on wedge/ring stimuli, the scotoma is concealed by the central clustering of pRF centers (bar: <2.5 degrees: 196 [9%], >2.5 degrees: 2090 [91%]; wedge/ring: <2.5 degrees 2358 [44%], and >2.5 degrees: 3022 [56%]) and at worst fails to reveal a scotoma. SMC values for this patient are 0.80 (<2.5 degrees) and 0.99 (>2.5 degrees) for bar stimuli and for wedge/ring stimuli 0.85 (<2.5 degrees) and 0.99 (>2.5 degrees), respectively. Due to a small shift in position of the scotoma, SMC values are smaller for the center of the visual field in maps made using bar stimuli even though it is entirely missing in the wedge/ring analogue.

Figures 3, 4, and 5 show data from three patients with GA. Each figure includes MP results superimposed on fundus photographs, pRF coverage maps calculated from fMRI data with bar and wedge/ring stimuli as well as binarized MP, and binarized pRF coverage maps with bar and wedge/ring stimuli. In addition, the pRF-based eccentricity map of the patient is presented overlaid on the white matter/grey matter (WM/GM) surface mesh.

### Quantitative Comparison

In a previous study, it was shown that wedge/ring compared to bar stimuli show improved fit in foveal areas up to about 2.5 degrees eccentricity.^22^ After binarizing the visual field maps and splitting them according to this 2.5 degrees boundary, SMCs can be compared not only between the stimuli but also between central and peripheral parts of the visual field. SMC values for the bar stimulus reached a mean value of 0.88 ± 0.13 for central areas and 0.88 ± 0.11 for peripheral areas, wedge/wing stimuli had a mean SMC value of 0.89 ± 0.12 in central areas and a mean SMC of 0.86 ± 0.10 for the peripheral visual field. Details appear in Figure 1.

The Wilcoxon signed rank was calculated across all subjects comparing SMC values for bar and wedge/ring stimuli for both the whole visual field as well as for inner (<2.5 degrees) and outer (>2.5 degrees) areas. There were no significant differences between SMC values for full visual field (*P* = 0.598) nor inner and outer subdivisions (<2.5 degrees: *P* = 0.317; >2.5 degrees: *P* = 0.786). Spearman rank-order correlation coefficient between the binarized coverage map values of pRF results from bar and wedge/ring stimuli showed a significant correlation (r = 0.499, *P* = < 0.05).

The distribution of pRF centers between inner and outer parts of the visual field was also expressed as a percentage of the total. On average, bar stimulus results had 40% of pRF centers located in the inner 2.5 degrees visual angle averaged over all patients compared to 53% when using wedge/ring stimuli. The difference was statistically significant (*P* = 0.012, paired *t*-test).

**Figure 6.**
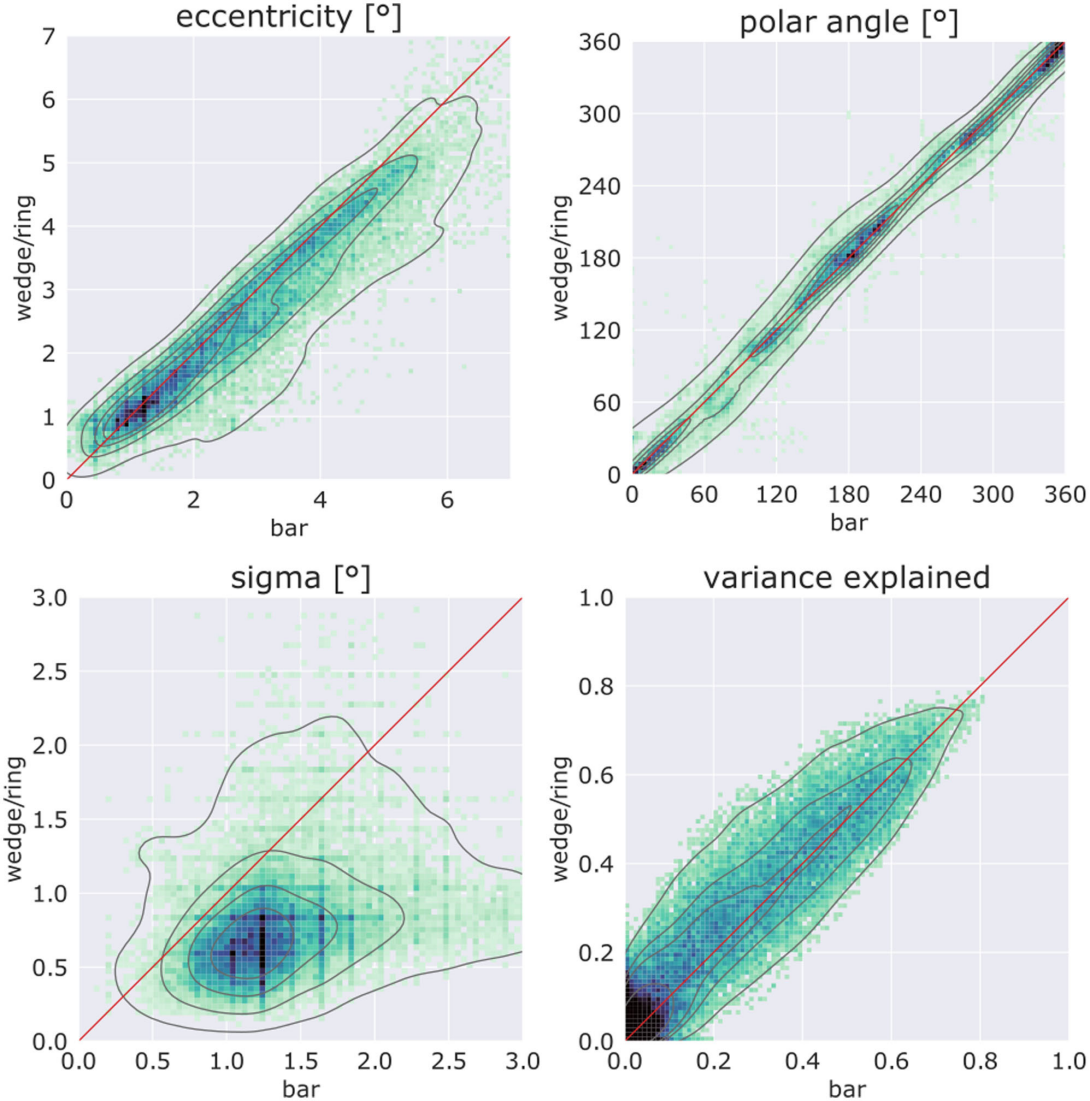
Point density plots comparing wedge/ring and bar results for eccentricity, polar angle, pRF size (sigma), and variance explained across all patients. Isodensity lines (grey, 20% steps) obtained from the initial point cloud and identity lines (*red*) are also shown.

This difference in distributions of pRF center positions between bar and wedge/ring stimuli was further examined based on eccentricity, polar angle, pRF size parameters, and variance explained values. The plots for these parameters including all patients’ V1 data points are displayed in Figure 6. Bar and wedge/ring values are plotted along the x- and y-axis, respectively. Due to the high number of points, initial point clouds were converted to histograms. In addition, isodensity lines (grey) obtained from the initial point cloud are presented. Points plotted on the 45 degrees line (red) indicate identical results in both analyses.

**Figure 7.**
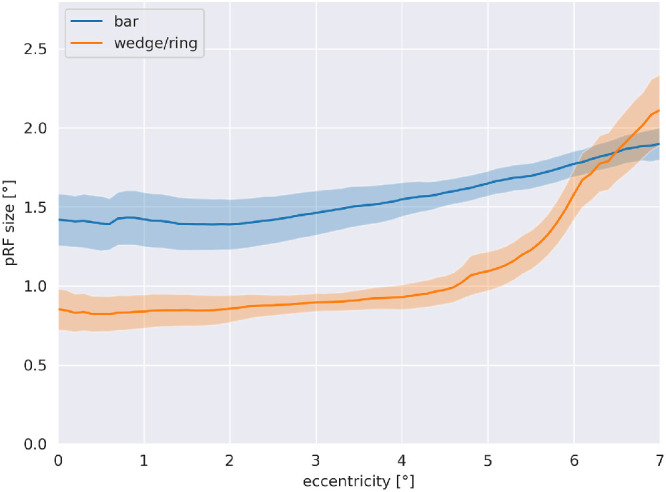
Relation of pRF size in regard to eccentricity.

Most eccentricity values fall below the red identity line, indicating lower eccentricity results using the wedge/ring stimulus (t(8) = 2.676, *P* = 0.028). In contrast, polar angles closely follow the red identity line, showing high similarity across both stimuli (t(8) = −0.226, *P* = 0.827). The third pRF parameter examined is size (sigma). Here, the discrepancy between pRF size parameters obtained from bar or wedge/ring stimulation is considerable. Most pRF size values fall below the red identity line demonstrating a clear, systemic bias of the wedge/ring stimulus towards smaller pRF size estimates when compared to bar stimuli (t(8) = 5.271, *P* = 0.001). Variance explained values show mostly similar results for the two stimuli, with a trend toward higher values in wedge/ring stimulation (t(8) = −1.878, *P* = 0.097). Relation of pRF size in regard to eccentricity can be seen in Figure 7; pRF sizes are stable within the central 4 degrees radius for both stimuli, however, showing higher values for the bar stimulus throughout the entire field of view. For the wedge/ring stimulus, the pRF size is strongly increased for the peripheral field of view.

## Discussion

The present study examines whether the choice of either bar or wedge/ring stimuli for pRF mapping in patients with GA secondary to AMD shows differences in distribution, position, or visual field coverage of pRF centers. All data were acquired using ultra-high-field MRI for maximum spatial resolution and sensitivity. Overall, analyses of pRF data for both stimuli show expected pRF size, eccentricity, and polar angle. The localized central macular dysfunction in the GA area with peripheral sparing was revealed by both stimuli at the cortical level, thereby complementing MP and retinal imaging (FAF and OCT).

Group-averaged SMC in patients with GA based on 7 Tesla fMRI data varied from 0.86 to 0.89 and corroborate previously published results where conventional test results, including MP and structural imaging, were linked to 3T fMRI based pRF-mapping of V1 in patients with Stargardt disease or retinitis pigmentosa.^4^ We utilized a similar methodology binarizing the coverage maps gained from MP and pRF mapping. This binarization is required as pRF mapping, in contrast to MP, uses only a single stimulus intensity and regions exhibiting low pRF coverage are therefore not equivalent to MP test points with low dB values. Coverage maps were also compared on a subject-by-subject basis and showed a high agreement. Although a number of studies have reported comparisons among retinal findings, visual fields, and fMRI results in a variety of visual pathway disorders, such as AMD, glaucoma or RP,^23^^–^^25^ this study is the first to compare different stimulus modalities in the evaluation of retinotopic features in patients with localized macular abnormalities.

Artificial scotomata were used in a previous study^5^ to simulate an unrealistically regular pattern of visual loss; artificial foveal scotomata with fixed location and size and sharp edges. This allowed for ground-truth conditions for exactly describing the effective retinal input generating the fMRI responses. Additionally, we showed that the presence of a central scotoma, even though it is a major disturbance in the model, does not negatively influence the pRF mapping results. In a different study, the influence of the stimulation paradigm on pRF results in healthy subjects showed systematic a bias toward more foveal pRF centers and lower pRF sizes using the wedge/ring stimulus.^22^ The findings further indicated that wedge/ring stimuli might be preferable in studies targeting the central visual field up to 2.5 degrees eccentricity, whereas bar stimuli could be advantageous in regions peripheral to that border. Herein, both approaches are used and applied to a real-world clinical setting with patients with GA having real patterns of visual loss, including scotomata of different sizes with irregular borders. Similar systematic biases were observed when comparing the stimuli, which confirms pRF mapping to be a robust and reliable method.

SMC calculation revealed no significant differences between stimulus variants, confirming pRF to be a robust approach for visual field mapping. Visual inspection of historical data from healthy subjects suggested a central clustering of pRF centers when using wedge/ring stimuli but a more homogenous distribution when using bar stimuli. This was confirmed by analysis of the percentage of pRF centers showing a statistically significant difference between stimulus variants in the distribution of pRF centers in inner and outer parts of the visual field. Further examination of pRF parameters revealed a bias of wedge/ring stimulus results toward lower eccentricity values. The size of pRFs shows a much stronger bias of the wedge/ring stimulus results toward markedly smaller pRF size estimates compared to bar stimulus results. In contrast, polar angles show high similarity across both stimuli. Table 3 shows that the variance explained threshold does exclude a slightly higher number of voxels with the bar compared to the wedge/ring stimulus, however, no significant difference was found. One explanation for the marked differences in pRF sizes between both stimulus variants might lie in the coverage of the bar stimulus compared to the wedge/ring stimulus. For the central visual field, only small areas are stimulated by the wedge/ring stimulus allowing for simultaneous viewing. This allows for mapping very small pRF sizes in foveal areas, whereas the bar stimulus has a continuous thickness throughout the whole visual field, possibly penalizing smaller pRFs. A further confounding factor is the difference of spatial characteristics of the checkerboard patterns. Although the bar stimulus exposes a rectangular checkerboard, wedge/ring stimuli expose radially symmetric checkerboards with a changing patch size with eccentricity. Previous publications try to adapt the stimulus regarding this problem by introducing eccentricity-scaled bar stimuli,^26^ however, these designs do not allow for a homogenous sampling of the visual field, which is of vast importance when examining patients with visual field loss.

**Table 3.** Distribution of pRF Centers Within the Visual Field

	Bar Stimulus			Wedge/Ring Stimulus		
	Total	<2.5 Degrees	>2.5 Degrees	Total	<2.5 Degrees	>2.5 Degrees
**Patient** **1**	1950	207 (10.6%)	1743 (89.4%)	4363	2351 (53.9%)	2012 (46.1%)
**Patient** **2**	3341	776 (23.2%)	2565 (76.8%)	4325	1515 (35.0%)	2810 (65.0%)
**Patient** **3**	6550	2640 (40.3%)	3910 (59.7%)	5932	2989 (50.4%)	2943 (49.6%)
**Patient** **4**	1843	768 (41.7%)	1075 (58.3%)	2259	1381 (61.1%)	878 (38.9%)
**Patient** **5**	2286	196 (8.6%)	2090 (91.4%)	5380	2358 (43.8%)	3022 (56.2%)
**Patient** **6**	7733	3628 (46.9%)	4105 (53.1%)	6992	3066 (43.9%)	3926 (56.1%)
**Patient** **8**	4428	3250 (73.4%)	1178 (26.2%)	4737	4011 (84.7%)	726 (15.3%)
**Patient 10**	6178	2371 (38.4%)	3807 (61.6%)	7086	3043 (42.9%)	4043 (57.1%)
**Patient 11**	4279	2382 (55.7%)	1897 (44.3%)	4612	2739 (59.4%)	1873 (40.6%)

Areas are subdivided into 0 to 2.5 degrees and 2.5 to 7 degrees based on the variance explained changing with increasing eccentricity based on a previous study.

The pRF sizes are stable within the central 4 degrees radius for both stimuli (see Fig. 7), however, showing higher values for the bar stimulus throughout the entire field of view. For the wedge/ring stimulus, the pRF size is strongly increased for the peripheral field of view, replicating previous findings in healthy subjects.^22^ There is one difference though: the steep slope toward the central visual field is missing. This could be explained by the fact that the measured scotomata are not taken into account in the model, leading to an increase in pRF sizes for central within scotoma pRFs.^20^

Data from individual patients showed stimulus-dependent differences between the binarized pRF maps. For example, the lower SMC value of bar stimuli maps of patient 5 indicates both the missing central scotoma (due to the slight misalignment of the scotoma in MP and the pRF map) and the immediately adjacent supposedly mistaken visual field defect (a consequence of the misalignment).

It has been shown that fMRI based pRF mapping is a highly stable method in healthy subjects^27^ and in subjects with simulated scotoma,^22^ in terms of pRF center position, and although we confirmed the stability when calculating SMC similarity of the two stimuli, there are significant differences in regard to pRF center distribution. At the same threshold of variance explained, wedge/ring stimuli show proportionately more above-threshold voxels in the central visual field. This distribution difference compared to bar results may arise from the changing size of the wedge/ring stimulus throughout the visual field (i.e. thickness changes from foveal to peripheral areas). This is likely to relate to the high cortical magnification factors of central regions and smaller pRF sizes.^28^ Subsequently, with changing pRF sizes in relation to eccentricity, larger parts of the peripheral visual field are exposed to wedge/ring stimuli compared with bar stimuli leading to less distinct stimulation and thus lower variance explained in voxels located in these areas.^22^ Size parameters have shown lower reproducibility in the literature, which might be explained by their strong dependency on the hemodynamic response function used for the analyses.^29^

The data from three initially recruited and scanned patients were excluded because of poor compliance in the MRI scanner (2 patients) or unstable fixation during the MP (1 patient). Both patients excluded for pRF instability demonstrated relatively unstable fixation on MP, suggesting fixation stability to be a major limiting factor in pRF mapping based on fMRI and that poor fixation greatly reduces the quality of pRF mapping results (see Table 3). The absence of eye-tracking at the 7 Tesla MRI scanner is a recognized limitation of the current study. A further limitation relates to the small field of view of the selected fMRI stimulus, due to the comparatively small bore of the 7T MRI scanner. Although the data contribute toward best practice methods in the use of fMRI in the assessment of patients with macular disease, the small sample size limits the ability to extrapolate to a larger patient population. Furthermore, due to the fact that no ground truth is known regarding pRF size, we cannot give a profound assessment of whether pRF sizes obtained using bar or wedge/ring stimuli are more neurophysiologically correct.

A major potential use for pRF mapping could be in the evaluation of patients receiving novel gene or cell replacement therapies, where functional gain of previously nonfunctional areas could be demonstrated objectively at the level of the visual cortex, as suggested by the recent data from patients with Leber congenital amaurosis (LCA).^11^ This method could provide additional quantitative biomarkers to serve as outcome measures augmenting existing clinical evaluations in novel treatment interventions.

One possible solution to compensate for the stimulus-specific coverage map differences would be a combination of multiple stimuli. Data have shown^22^ that the combination of pRF mapping data unsurprisingly improves the homogeneity of coverage map results. Combining multiple runs further effectively compensates the stimulus-specific deficits (i.e. pRF center distribution) in visual field coverage. It may also be of interest in future studies to concentrate upon the distribution of pRF centers as opposed to pRF size when interpreting visual field maps. A perfect example for this phenomenon would be the maps of patient 3 where the scotoma is demarcated in great detail irrespective of the stimulus used but occluded by pRF size on visual field maps.

## Conclusions

The present study further confirms pRF mapping to be a robust and reliable method and demonstrates that while bar and wedge/ring stimuli may show significant differences in the distribution of pRF centers across the visual field, this variability has minimal impact on the comparison with microperimetry. The demonstrated differences in pRF mapping results consequent upon the choice of visual stimulus needs adequate consideration if pRF mapping is to be used in a clinical setting. The selection of the most appropriate visual stimulus for assessment of visual cortical function in retinal disease needs to be based upon established stimulus-specific relationships between dysfunction in defined retinal regions and activity in the corresponding cortical areas. Future studies that focus on comparison measures beyond the SMC may further ascertain and quantify the characteristics of individual stimulus modalities.

## References

[1] Wandell BA, Dumoulin SO, Brewer AA. Visual field maps in human cortex. *Neuron*. 2007; 56(2): 366–383. 17964252 10.1016/j.neuron.2007.10.012

[2] Wandell BA, Winawer J. Computational neuroimaging and population receptive fields. *Trends Cogn Sci*. 2015; 19(6): 349–357. 25850730 10.1016/j.tics.2015.03.009 PMC4484758

[3] Dumoulin SO, Wandell BA. Population receptive field estimates in human visual cortex. *Neuroimage*. 2008; 39(2): 647–660. 17977024 10.1016/j.neuroimage.2007.09.034 PMC3073038

[4] Ritter M, Hummer A, Ledolter AA, Holder GE, Windischberger C, Schmidt-Erfurth UM. Correspondence between retinotopic cortical mapping and conventional functional and morphological assessment of retinal disease. *Br J Ophthalmol*. 2019; 103(2): 208–215. 29699983 10.1136/bjophthalmol-2017-311443

[5] Hummer A, Ritter M, Woletz M, et al. Artificial scotoma estimation based on population receptive field mapping. *Neuroimage*. 2018; 169: 342–351. 29253656 10.1016/j.neuroimage.2017.12.010

[6] Sunness JS, Liu T, Yantis S. Retinotopic mapping of the visual cortex using functional magnetic resonance imaging in a patient with central scotomas from atrophic macular degeneration. *Ophthalmology*. 2004; 111(8): 1595–1598. 15288993 10.1016/j.ophtha.2003.12.050

[7] Baseler HA, Gouws A, Crossland MD, et al. Objective visual assessment of antiangiogenic treatment for wet age-related macular degeneration. *Optom Vis Sci*. 2011; 88(10): 1255–1261. 21705938 10.1097/OPX.0b013e3182282f13

[8] Silson EH, Aleman TS, Willett A, et al. Comparing clinical perimetry and population receptive field measures in patients with choroideremia. *Investig Ophthalmol Vis Sci*. 2018; 59(8): 3249–3258. 29971442 10.1167/iovs.18-23929 PMC6110169

[9] Ashtari M, Cyckowski L, Yazdi A, et al. fMRI of retina-originated phosphenes experienced by patients with Leber congenital amaurosis. *PLoS One*. 2014; 9(1): e86068. 24465873 10.1371/journal.pone.0086068 PMC3897613

[10] Papanikolaou A, Keliris GA, Papageorgiou TD, et al. Population receptive field analysis of the primary visual cortex complements perimetry in patients with homonymous visual field defects. *Proc Natl Acad Sci USA*. 2014; 111(16): E1656–1665. 24706881 10.1073/pnas.1317074111 PMC4000790

[11] Ashtari M, Nikonova ES, Marshall KA, et al. The Role of the Human Visual Cortex in Assessment of the Long-Term Durability of Retinal Gene Therapy in Follow-on RPE65 Clinical Trial Patients. *Ophthalmology*. 2017; 124(6): 873–883. 28237426 10.1016/j.ophtha.2017.01.029 PMC5805133

[12] Engel SA, Rumelhart DE, Wandell BA, et al. FMRI of human visual cortex. *Nature*. 1994; 369(6481): 525. 8031403 10.1038/369525a0

[13] Sereno MI, Dale AM, Reppas JB, et al. Borders of multiple visual areas in humans revealed by functional magnetic resonance imaging. *Science*. 1995; 268(5212): 889–893. 7754376 10.1126/science.7754376

[14] Infanti E, Schwarzkopf DS. Mapping sequences can bias population receptive field estimates. *Neuroimage*. 2020; 211: 116636. 32070751 10.1016/j.neuroimage.2020.116636

[15] Yildirim F, Carvalho J, Cornelissen FW. A second-order orientation-contrast stimulus for population-receptive-field-based retinotopic mapping. *Neuroimage*. 2018; 164: 183–193. 28666882 10.1016/j.neuroimage.2017.06.073

[16] Ahmadi K, Herbik A, Wagner M, Kanowski M, Thieme H, Hoffmann MB. Population receptive field and connectivity properties of the early visual cortex in human albinism. *Neuroimage*. 2019; 202: 116105. 31422172 10.1016/j.neuroimage.2019.116105

[17] Liu T, Cheung SH, Schuchard RA, et al. Incomplete cortical reorganization in macular degeneration. *Invest Ophthalmol Vis Sci*. 2010; 51(12): 6826–6834. 20631240 10.1167/iovs.09-4926 PMC3055781

[18] Fleckenstein M, Mitchell P, Freund KB, et al. The Progression of Geographic Atrophy Secondary to Age-Related Macular Degeneration. *Ophthalmology*. 2018; 125(3): 369–390. 29110945 10.1016/j.ophtha.2017.08.038

[19] Linhardt D, Pawloff M, Woletz M, et al. Intrasession and Intersession Reproducibility of Artificial Scotoma pRF Mapping Results at Ultra-High Fields. *eNeuro*. 2022; 9(5): ENEURO.0087–22.2022. 36635900 10.1523/ENEURO.0087-22.2022 PMC9512620

[20] Binda P, Thomas JM, Boynton GM, Fine I. Minimizing biases in estimating the reorganization of human visual areas with bold retinotopic mapping. *J Vis*. 2013; 13(7): 13 10.1167/13.7.13 PMC3689563 23788461

[21] Moeller S, Yacoub E, Olman CA, et al. Multiband multislice GE-EPI at 7 tesla, with 16-fold acceleration using partial parallel imaging with application to high spatial and temporal whole-brain fMRI. *Magn Reson Med*. 2010; 63(5): 1144–1153. 20432285 10.1002/mrm.22361 PMC2906244

[22] Linhardt D, Pawloff M, Hummer A, et al. Combining stimulus types for improved coverage in population receptive field mapping. *Neuroimage*. 2021; 238: 118240. 34116157 10.1016/j.neuroimage.2021.118240

[23] Ferreira S, Pereira AC, Quendera B, Reis A, Silva ED, Castelo-Branco M. Primary visual cortical remapping in patients with inherited peripheral retinal degeneration. *NeuroImage Clin*. 2016; 13: 428–438. 28116235 10.1016/j.nicl.2016.12.013 PMC5233796

[24] Goesaert E, Van Baelen M, Spileers W, Wagemans J, Op De Beeck HP. Visual space and object space in the cerebral cortex of retinal disease patients. *PLoS One*. 2014; 9(2): e88248. 24505449 10.1371/journal.pone.0088248 PMC3914958

[25] Baseler HA, Gouws A, Haak K V., et al. Large-scale remapping of visual cortex is absent in adult humans with macular degeneration. *Nat Neurosci*. 2011; 14(5): 649–655. 21441924 10.1038/nn.2793

[26] Alvarez I, de Haas B, Clark CA, Rees G, Samuel Schwarzkopf D. Comparing different stimulus configurations for population receptive field mapping in human fMRI. *Front Hum Neurosci*. 2015; 9: 96. 25750620 10.3389/fnhum.2015.00096 PMC4335485

[27] van Dijk JA, de Haas B, Moutsiana C, Schwarzkopf DS. Intersession reliability of population receptive field estimates. *Neuroimage*. 2016; 143: 293–303. 27620984 10.1016/j.neuroimage.2016.09.013 PMC5139984

[28] Harvey BM, Dumoulin SO. The relationship between cortical magnification factor and population receptive field size in human visual cortex: Constancies in cortical architecture. *J Neurosci*. 2011; 31(38): 13604–13612. 21940451 10.1523/JNEUROSCI.2572-11.2011 PMC6623292

[29] Lerma-Usabiaga G, Benson N, Winawer J, Wandell BA. A validation framework for neuroimaging software: The case of population receptive fields. *PLoS Comput Biol*. 2020; 16(6): e1007924. 32584808 10.1371/journal.pcbi.1007924 PMC7343185

